# From Prediction to Monitoring: Toward a Translational Framework of Biomarkers in Spinal Cord Stimulation

**DOI:** 10.3390/biomedicines14061307

**Published:** 2026-06-09

**Authors:** Gustavo Fabregat-Cid, Natalia Escrivá-Matoses, José De Andrés

**Affiliations:** 1 Multidisciplinary Pain Management Department, Hospital General Universitario de Valencia, 46014 Valencia, Spain; 2 Department of Surgery, Universitat de València, 46010 Valencia, Spain; 3 Generalitat Valenciana, 46001 Valencia, Spain; escriva_nat@gva.es

**Keywords:** spinal cord stimulation, biomarkers, neuromodulation, predictive biomarkers, monitoring biomarkers, electroencephalography, functional neuroimaging, chronic pain, precision medicine

## Abstract

Spinal cord stimulation (SCS) is an established therapy for chronic pain, yet treatment response remains highly variable and patient selection largely empirical. The identification of biomarkers with the potential to predict and monitor therapeutic response is therefore critical for advancing toward precision neuromodulation. This study provides a structured narrative synthesis of current evidence on biomarkers in SCS, focusing on their predictive and monitoring roles and their translational potential. Available studies were analysed across electrophysiological, neuroimaging, autonomic, and molecular domains and conceptually organized into predictive biomarkers—reflecting baseline biological states associated with treatment susceptibility—and monitoring biomarkers, capturing physiological and molecular adaptations following stimulation. Among predictive approaches, intraoperative electroencephalography (EEG) and resting-state functional magnetic resonance imaging (rs-fMRI) have shown promising but exploratory discriminative performance. However, EEG findings are derived from intraoperative settings, limiting their applicability to pre-implantation patient selection. In contrast, monitoring biomarkers—including heart rate variability, metabolic imaging, and immunological parameters—provide objective measures of treatment-induced changes but do not currently support predictive use. Molecular and genomic biomarkers, while mechanistically informative, remain exploratory and lack validated clinical utility. A central limitation of the field is the fragmentation of biomarker research, with most studies evaluating single modalities in isolation. To address this gap, we propose a translational framework integrating predictive and monitoring biomarkers through a two-stage model combining baseline stratification with longitudinal response assessment. Although biomarker research in SCS is rapidly evolving, its clinical application remains limited. The development of multimodal, validated biomarker strategies may support improved patient selection and more objective evaluation of treatment response, enabling a transition toward mechanism-based neuromodulation.

## 1. Introduction

Chronic pain represents a major global health burden, affecting approximately 20% of the adult population and significantly impairing quality of life, functional capacity, and socioeconomic outcomes [1]. Its multifactorial nature—encompassing biological, psychological, and social dimensions—limits the utility of purely subjective assessment tools and complicates therapeutic decision-making [2]. In this context, spinal cord stimulation (SCS) has emerged as an established treatment for refractory chronic pain conditions such as persistent spinal pain syndrome type 2 (PSPS-T2), complex regional pain syndrome (CRPS), and painful diabetic neuropathy, demonstrating sustained improvements in pain intensity, disability, and quality of life in selected patients [3,4,5,6].

However, despite its proven efficacy at the population level, individual response to SCS remains highly variable, with a substantial proportion of patients failing to achieve clinically meaningful benefit [3,7]. Current patient selection relies largely on clinical evaluation and trial stimulation, which are inherently subjective and resource-intensive [8]. This variability highlights a critical unmet need: the identification of objective biomarkers capable of predicting treatment response, monitoring therapeutic effects, and providing mechanistic insights into neuromodulation.

In recent years, increasing attention has been directed toward the development of biomarkers in pain medicine [9]. These encompass a wide range of modalities, including neuroimaging techniques such as functional magnetic resonance imaging (fMRI) and positron emission tomography (PET), electrophysiological measures such as electroencephalography (EEG) and magnetoencephalography (MEG), physiological markers such as heart rate variability (HRV), and molecular approaches including cytokine profiling, proteomics, and gene expression analyses [10,11,12,13,14,15,16,17,18]. Each of these domains offers a distinct perspective on the neurobiological substrates of pain and its modulation through SCS.

Neuroimaging studies have suggested that functional connectivity within key regions of the pain matrix, including the anterior cingulate cortex and default mode network, may be associated with differential response to neuromodulation [11]. Similarly, electrophysiological approaches have demonstrated that specific cortical oscillatory patterns, particularly within theta and alpha frequency bands, may reflect pain states and potentially predict treatment outcomes [17]. Parallel to these developments, molecular studies have identified changes in inflammatory mediators, immune cell populations, and gene expression profiles following SCS, supporting the concept that neuromodulation exerts systemic neuroimmune effects beyond local spinal mechanisms [19,20].

Despite these advances, the current evidence remains fragmented and heterogeneous. Most studies are characterized by small sample sizes, lack of external validation, and variability in methodological approaches. Furthermore, the distinction between predictive biomarkers (capable of identifying responders prior to treatment), monitoring biomarkers (reflecting dynamic changes during therapy), and mechanistic biomarkers (providing biological insights without immediate clinical applicability) is often unclear or inconsistently applied. This lack of conceptual clarity limits the translation of biomarker research into clinical practice.

Therefore, a structured synthesis of the available evidence is needed, not only to summarize existing findings but also to critically appraise the level of evidence and clinical applicability of different biomarker categories. The aim of this review is to provide a comprehensive and critical overview of biomarkers associated with spinal cord stimulation in chronic pain, with a specific focus on distinguishing predictive, monitoring, and mechanistic biomarkers. By clarifying the current state of the field and identifying key gaps in the literature, this work seeks to inform future research and contribute to the development of precision medicine approaches in neuromodulation.

## 2. Materials and Methods

This study was designed as a structured narrative review incorporating a systematic search strategy. Although a systematic search strategy was applied to identify relevant studies, the objective was to provide a focused synthesis of key evidence and to develop a translational framework rather than to perform a formal systematic review or meta-analysis.

### 2.1. Search Strategy

A structured search was conducted in PubMed using the following query: (“spinal cord stimulation” OR “SCS”) AND (“biomarker” OR “biomarkers” OR “EEG” OR “electroencephalography” OR “functional MRI” OR “fMRI” OR “PET” OR “heart rate variability” OR “HRV” OR “cytokines” OR “proteomics” OR “genomics” OR “transcriptomics”) AND (“prediction” OR “predictive” OR “response” OR “outcome” OR “monitoring”).

The search was restricted to:

- Human studies.
- Articles published in English.
- Studies reporting original data.

No strict date limits were imposed, although emphasis was placed on contemporary studies reflecting current SCS technologies and biomarker methodologies.

### 2.2. Eligibility Criteria

Studies were included if they met the following criteria:

1. Investigated patients undergoing spinal cord stimulation for chronic pain conditions (e.g., PSPS-T2, CRPS, neuropathic pain).
2. Evaluated at least one biomarker, defined as a measurable physiological, imaging, or molecular parameter.
3. Reported an association between the biomarker and the following:
  ○ Treatment response;
  ○ Clinical outcomes (e.g., pain intensity, disability);
  ○ Or biological effects of SCS.

Exclusion criteria were:

- Studies without objective biomarkers (e.g., purely clinical or patient-reported predictors without biological measures).
- Technical or engineering studies without clinical correlation.
- Review articles, editorials, and guidelines.
- Case reports.
- Studies not specifically addressing spinal cord stimulation.

### 2.3. Study Selection

Study selection was performed through a two-step screening process:

1. Title and abstract screening, excluding clearly irrelevant studies.
2. Full-text assessment of potentially eligible articles.

Study selection was performed through consensus-based evaluation.

A pragmatic approach was adopted to prioritize studies with clear clinical or translational relevance. Given the heterogeneity of the field and the exploratory nature of many biomarker studies, emphasis was placed on methodological clarity and the presence of clinically meaningful outcomes rather than strict methodological homogeneity.

In addition, one relevant study identified through reference list screening of relevant articles and not retrieved through the initial PubMed search was included after full-text assessment to ensure completeness of the evidence synthesis.

**Figure 1:**
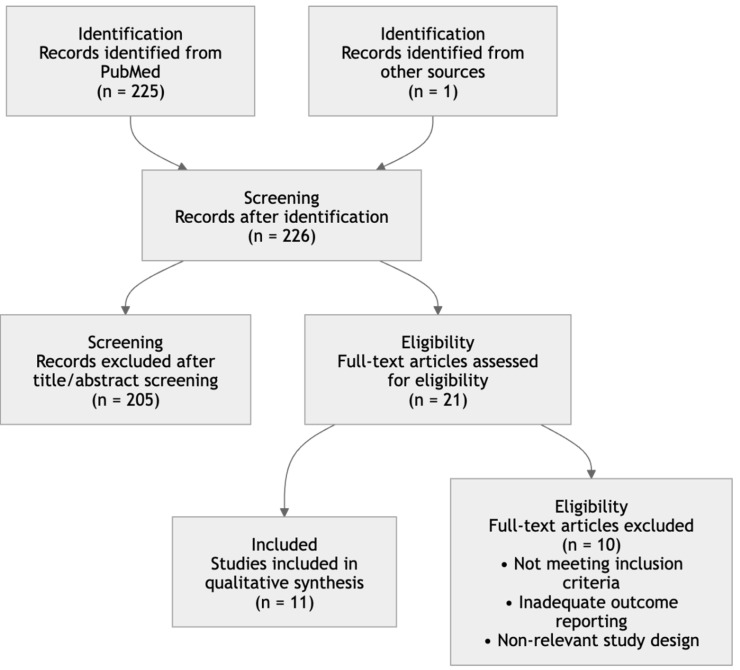
Flow diagram of the study selection process. A total of 226 records were identified (225 through PubMed and 1 from other sources). After title and abstract screening, 205 records were excluded. In total, 21 full-text articles were assessed for eligibility, of which 10 were excluded for not meeting inclusion criteria, inadequate outcome reporting, or non-relevant study design. Ultimately, 11 studies were included in the qualitative synthesis.

A simplified flow diagram was constructed to illustrate the study selection process (Figure 1).

### 2.4. Data Extraction

Data from included studies were systematically extracted and organized into a structured evidence table (Table 1). The following variables were collected:

- Study design.
- Population characteristics and sample size.
- Type of biomarker (neuroimaging, electrophysiological, molecular, physiological).
- Specific biomarker parameters.
- Timing of measurement (pre-implant, intraoperative, post-implant, longitudinal).
- Clinical outcomes.
- Key quantitative findings (e.g., AUC, accuracy, correlations).
- Study limitations.

**Table 1:** Summary of included studies evaluating biomarkers in spinal cord stimulation (SCS). Studies are categorized according to biomarker type, study design, and clinical relevance.

Reference	First Author, Year	Study Design	Population (N, Condition)	Biomarker Type	Specific Biomarker	Timing (Baseline vs. Post-SCS)	Outcome	Key Findings (Quantitative If Available)	Classification (Predictive/Monitoring/Mechanistic)	Level of Evidence
[16]	De Andrés, 2021	Prospective longitudinal study	N = 16, FBSS	Genomic/Protein (PBMCs)	PENK, CB1, CB2, IL-1 b gene/protein expression	Baseline vs. 24 h, 5 d, 15 d, 2 m	NRS, PD-Q, ODI, SF-12	PENK significantly increased (*p* = 0.000; median increase 3.22). PENK changes correlated positively with VAS and negatively with SF-12 MCS. IL-1 b correlated negatively with PD-Q.	Mechanistic/Monitoring	Level II
[14]	Fabregat-Cid, 2023	Prospective case–control study	N = 30 PSPS-2, N = 14 HC	Serum Proteomics	462 proteins (Mass Spectrometry)	Baseline vs. 2 w, 2 m, 6 m, 12 m	NRS, ODI, PD-Q, HADS, SF-12	Responders showed downregulation of immune/inflammatory proteins and upregulation of synaptic/metabolic proteins. 83 proteins remained significant throughout (*p* ≤ 0.05).	Mechanistic (with potential predictive value)	Level II
[15]	Fabregat-Cid, 2024	Prospective GWAS	N = 30 PSPS-2, N = 15 HC	Genomic (mRNA Microarray)	mRNA expression profiling (microarray)	Baseline vs. 2 w, 2 m	NRS, PD-Q, ODI, HADS, SF-12	11 genes downregulated in PSPS-2 vs. HC. 2 genes (FUT6, TNS2-AS1) downregulated post-SCS response. Evidence of enhanced inflammation/proliferative response in PSPS-2.	Mechanistic	Level II
[21]	Gopal, 2025	Prospective cohort/ML validation	N = 20, Chronic pain (PSPS, CRPS, CLBP)	Electrophysiological (EEG)	Intraoperative EEG features (including alpha/theta ratio)	Intraoperative Baseline vs. Tonic vs. HD	≥50% NRS reduction at 3 m	ML model predicted responders with 88.2% accuracy. Alpha-theta ratio differed by region (*p* = 0.019). Baseline FP1-FP2 ratio correlated with NRS (r = −0.800, *p* = 0.041).	Predictive	Level II
[12]	Goudman, 2021	Prospective comparative study	N = 22, FBSS	Physiological (Autonomic)	HRV indices (IBI, HR, SDNN, RMSSD, LF, HF) via ECG	Baseline (12 h off) vs. 40 min post-SCS	Autonomic balance/Device reliability	Significant increase in IBI (*p* = 0.001) and absolute HF power (*p* = 0.01); decrease in HR (*p* = 0.001) and normalized LF (*p* = 0.02) during SCS. Good agreement between measurement methods (r ≥ 0.82).	Monitoring/Mechanistic	Level II-2
[22]	Kinfe, 2017	Prospective feasibility cohort	N = 12, FBSS	Plasma Cytokines	IL-10, HMGB1, IL-1 b, TNF-a	Baseline vs. 3 months (Burst SCS)	VAS, PSQI, BDI	IL-10 significantly elevated post-SCS (43.16 vs. 12.54 pg/mL; *p* = 0.03). Pre-burst IL-10 correlated with VAS-B (r = −0.66). Post-burst IL-10 correlated with PSQI (r = −0.66).	Mechanistic/Monitoring	Level II
[13]	Kogias, 2025	Prospective cohort study	N = 16 PSPS-T2, N = 16 HC	Cytometry/Cytokine Multiplex	Plasma cytokines; T-lymphocyte subsets (TH17, NKT, TEMRA)	Baseline vs. 7–10 days (10 kHz)	NRS, DASS42, PCS, NPQ	Significant reduction in IL-1 b, IL-6, IL-8, IL-10, IL-12, and IFN-g. Reduction in TH17 and NKT abundance correlated with pain relief (*p* < 0.05).	Mechanistic/Monitoring	Level II
[10]	Poply, 2023	Randomized blinded crossover trial	N = 14, FBSS (Intractable lumbar neuropathic pain)	Metabolic (Imaging)	18F-FDG PET-CT (SUVmax)	Baseline vs. 4 weeks (40 Hz, 4 kHz, 10 kHz)	NRS, ODI, EQ-5D-5L, PGIC	Significant SUVmax reduction at 40 Hz (*p* = 0.002) and 4 kHz (*p* = 0.001). Thalamic metabolic reduction reached 59.5%. 10 kHz PET uptake correlated with NRS-L (*p* = 0.011).	Mechanistic	Level II
[23]	Telkes, 2020	Prospective intraoperative study	N = 9, FBSS or CLBP	Electrophysiological (EEG)	Spectral EEG (Alpha power, Peak power ratio)	Intraoperative Baseline vs. Tonic vs. 10 kHz	NRS, ODI at 3 months	10 kHz SCS increased S1 relative alpha power (*p* = 0.005) and shifted peak from theta to alpha. Alpha/theta ratio in S1/Frontal regions strong correlation with ODI change (Spearman r = 1.00, *p* = 0.017; small sample).	Monitoring/Mechanistic	Level II
[11]	Ueno, 2025	Prospective clinical investigation	N = 29, Chronic pain (PSPS-2, CRPS, PHN)	Neurological (Imaging)	rs-fMRI (Functional Connectivity)	Baseline (pre-SCS trial)	≥50% NRS reduction (Trial responsiveness)	Mid ACC-precuneus/PCC connectivity was significantly lower in responders. Area under the curve (AUC) = 0.814, sensitivity 71%, specificity 87% (*p* < 0.001).	Predictive	Level II-2
[24]	Witjes, 2025	Case–control/ML classification	N = 75 (25 chronic pain, 25 SCS, 25 HC)	Neurological (MEG)	MEG spectral features (Theta, Alpha, Beta, Low-gamma)	Post-SCS (Tonic, Burst, Sham)	Pain classification/Treatment effect	Theta/alpha power ratio classified chronic pain with 76% accuracy (AUC 0.80). Poor correlation (rho = 0.12) between model output and SCS pain scores.	Mechanistic (diagnostic classification)	Level II-2

This structured approach enabled comparison across heterogeneous study designs and biomarker modalities.

### 2.5. Biomarker Classification

To facilitate interpretation, biomarkers were classified into three categories based on their clinical role:

- Predictive biomarkers: measured before or during implantation and associated with subsequent treatment response.
- Monitoring biomarkers: reflecting dynamic changes during or after SCS.
- Mechanistic biomarkers: providing insight into biological processes without demonstrated clinical predictive utility.

This classification framework is consistent with established concepts in biomarker research and translational medicine, distinguishing between exploratory biological signals and clinically actionable tools [25,26].

Given the overlap between biological interpretation and clinical applicability, mechanistic biomarkers were incorporated within predictive or monitoring sections when appropriate.

### 2.6. Evidence Appraisal

Given the heterogeneity of study designs and the absence of standardized methodologies across biomarker studies in SCS, a formal meta-analysis was not performed. Instead, a qualitative critical appraisal was conducted.

Each study was evaluated according to:

- Sample size.
- Study design (prospective vs. retrospective).
- Presence of quantitative predictive metrics (e.g., AUC, accuracy).
- External validation.

Based on these criteria, studies were categorized according to a simplified hierarchical framework adapted from established evidence classification systems, and their clinical applicability was assessed in terms of feasibility and potential for implementation in routine clinical practice.

## 3. Predictive Biomarkers

The main characteristics, biomarker domains, and key findings of the included studies are summarized in Table 1.

The identification of predictive biomarkers in spinal cord stimulation (SCS) aims to optimize patient selection using electrophysiological and baseline neuroimaging tools. Among the included studies, evidence supporting predictive capability is limited to a small number of approaches, while other biomarker domains remain exploratory and lack validated predictive performance.

### 3.1. Electrophysiological Biomarkers

Electrophysiological approaches, particularly intraoperative electroencephalography (EEG), have been explored as potential tools to identify neurophysiological signatures associated with response to spinal cord stimulation (SCS).

In an exploratory study, machine learning models applied to intraoperative EEG signals demonstrated the ability to differentiate responders from non-responders at 3 months (defined as ≥50% reduction in NRS), achieving an accuracy of 88.2% and an area under the curve (AUC) of 0.879 [21]. However, these findings were obtained in a small exploratory cohort without independent external validation, limiting their generalizability. In this context, machine learning performance metrics should be interpreted cautiously, as high-dimensional electrophysiological features combined with limited sample sizes increase the risk of overfitting and optimistic model performance estimates. Furthermore, as these data are acquired intraoperatively, their applicability to pre-implantation patient selection remains limited.

Beyond predictive modelling, intraoperative EEG features have been associated with clinical outcomes. Spectral characteristics such as theta peak frequency and stimulation-induced modulation of alpha activity have been identified as relevant markers of cortical dynamics during neuromodulation [21,23]. In addition, the alpha/theta peak power ratio in frontal and somatosensory regions has shown associations with functional improvement, as measured by the Oswestry Disability Index (ODI); however, these findings are based on a very small sample and should be interpreted with caution as exploratory observations requiring validation in larger cohorts [23].

Magnetoencephalography (MEG) has also been investigated as a method to characterize brain activity in chronic pain. Models based on oscillatory activity, including theta power and alpha power ratios, have demonstrated moderate accuracy in distinguishing patients with chronic pain, patients treated with SCS, and healthy controls [24]. However, these findings primarily reflect pain-state classification rather than prediction of response to SCS. Therefore, MEG should be considered a mechanistic or exploratory biomarker rather than a predictive tool for treatment response.

### 3.2. Neuroimaging Biomarkers

Functional neuroimaging using resting-state functional magnetic resonance imaging (rs-fMRI) has provided insights into the network-level mechanisms underlying response to spinal cord stimulation (SCS). Rather than isolated regional activity, these approaches emphasize alterations in large-scale brain networks involved in pain processing and modulation.

In a prospective cohort study, baseline functional connectivity between the mid-anterior cingulate cortex (mid-ACC) and posterior default mode network regions (praecuneus/posterior cingulate cortex) differentiated responders from non-responders [11]. Responders exhibited lower baseline connectivity within this circuit, whereas higher connectivity was observed in non-responders, indicating an inverse relationship between connectivity strength and treatment response. Moderate discriminative performance (AUC 0.814; sensitivity 71%; specificity 87%) was observed in a single-cohort analysis [11].

Beyond this specific finding, emerging evidence suggests that treatment response to SCS may be influenced by the functional organization of large-scale brain networks. Interactions between the anterior cingulate cortex, default mode network, and salience network are consistently implicated in pain modulation across studies [27,28,29]. These networks are involved in integrating sensory, cognitive, and affective dimensions of pain, and their dysregulation has been consistently described in chronic pain conditions [28].

Within this framework, SCS has been proposed to exert its therapeutic effects not only through spinal mechanisms but also by modulating connectivity between somatosensory regions and higher-order networks involved in attention, emotion, and self-referential processing. This network-based perspective may help explain inter-individual variability in treatment response, raising the hypothesis that patients with more preserved or flexible connectivity patterns may be more responsive to neuromodulation, although this remains to be validated.

### 3.3. Other Biomarker Domains

Within the included studies, other biomarker modalities, including molecular and genomic approaches, did not demonstrate validated predictive capability.

Serum proteomic analysis identified baseline differences in the expression of proteins involved in inflammatory, immune, and coagulation pathways between patient phenotypes [14]. However, these findings were not associated with validated predictive models or reproducible performance metrics suitable for clinical application and are better interpreted as reflecting underlying disease biology rather than established predictive biomarkers.

Similarly, genomic and transcriptomic studies identified alterations in the expression of genes related to neuroinflammatory processes. Changes in biomarkers such as proenkephalin (PENK) were associated with clinical evolution following implantation, reflecting post-treatment biological responses rather than baseline predictive capacity [15,16], including correlations with pain intensity and quality-of-life measures across longitudinal assessments. Accordingly, these markers are better interpreted as monitoring or mechanistic biomarkers rather than predictive tools.

## 4. Monitoring Biomarkers

Monitoring of response to spinal cord stimulation (SCS) relies on the assessment of physiological, metabolic, and immunological changes following device activation. In contrast to predictive biomarkers, these measures reflect dynamic adaptations to neuromodulation rather than baseline susceptibility. The main characteristics and findings of monitoring biomarker studies are summarized in Table 1.

### 4.1. Autonomic Biomarkers

Heart rate variability (HRV) has been used as a physiological marker of autonomic response to SCS. Activation of SCS has been associated with increases in interbeat interval (IBI) and high-frequency (HF) oscillations, suggesting a shift toward parasympathetic dominance [12]. These changes have been detected using wearable devices, which demonstrate strong agreement with standard electrocardiographic measurements, supporting their feasibility for ambulatory monitoring [12]. However, these observations are based on short-term measurements, and their applicability for longitudinal monitoring remains to be established.

### 4.2. Neuroimaging Biomarkers

Functional and metabolic neuroimaging techniques have been used to characterize treatment-induced changes in brain activity. 18F-FDG PET imaging has shown reductions in metabolic activity within key regions of the pain matrix, including the thalamus, primary somatosensory cortex, and insula following SCS [10]. These findings are consistent with modulation of central pain processing; however, they should be interpreted as primarily mechanistic, and their relationship with clinically actionable monitoring remains limited. These effects appear to be frequency-dependent, with significant reductions observed at 40 Hz and 4 kHz, whereas at 10 kHz stimulation, metabolic changes were less consistent and primarily correlated with clinical outcomes rather than showing uniform reductions.

### 4.3. Immunological and Molecular Biomarkers

SCS has been associated with measurable changes in systemic immune and inflammatory profiles. Reductions in pro-inflammatory cytokines (e.g., IL-1β, IL-6, IL-8, IL-12, IFN-γ) and changes in immune cell populations, including TH17 and NKT cells, have been reported following stimulation [13]. In addition, modulation of anti-inflammatory cytokines such as IL-10 appears to be dependent on the stimulation paradigm, with increases reported in burst SCS associated with improvements in sleep quality [22], while reductions have been observed under high-frequency 10 kHz stimulation [13].

At the molecular level, proteomic analyses suggest partial normalization of baseline inflammatory signatures following SCS [14]. Genomic studies have identified alterations in pathways related to neuroinflammation, although no consistent predictive signatures have been validated [15]. Taken together, these findings support a primarily mechanistic role, with potential but unproven predictive relevance.

In addition, gene expression studies have reported increased proenkephalin (PENK) mRNA levels in peripheral blood mononuclear cells, which have been associated with variations in pain intensity across longitudinal assessments following implantation [16]. Overall, these findings remain exploratory and require validation.

## 5. Translational Integration

The translation of biomarker research into clinical practice in spinal cord stimulation (SCS) remains limited, despite increasing evidence across electrophysiological, neuroimaging, autonomic, and molecular domains [11,12,14,16,21,30,31]. The primary challenge is not the identification of candidate biomarkers, but their integration into clinically actionable frameworks that can support patient selection, guide therapy optimization, and enable objective monitoring of treatment response.

Across the available literature, a distinction can be made between predictive biomarkers, which reflect baseline neurobiological states associated with treatment susceptibility, and monitoring biomarkers, which capture physiological and molecular adaptations following stimulation. Electrophysiological and neuroimaging approaches, particularly intraoperative EEG and resting-state functional connectivity, have demonstrated potential for patient stratification, although evidence remains limited to small, exploratory cohorts [11,21]. However, intraoperative biomarkers, while potentially informative, do not address the need for pre-implantation stratification. In contrast, autonomic, metabolic, and immunological markers provide objective measures of treatment-induced changes but have not shown consistent predictive utility [12,13,22].

These findings align with contemporary models of chronic pain as a disorder of large-scale brain networks, involving alterations in connectivity and central processing mechanisms [32,33]. Within this context, baseline biomarkers may be conceptualized as potential indicators of neural susceptibility to modulation, whereas dynamic biomarkers reflect the biological response to neuromodulation across central and peripheral systems, including autonomic and neuroimmune pathways [14,20,21].

Beyond their potential predictive or monitoring roles, mechanistic biomarkers may also contribute to the biological characterization of chronic pain phenotypes. Neuroimmune, autonomic, and network-level biomarkers could help identify distinct pathophysiological profiles, including predominant neuroinflammatory states, central sensitization patterns, or maladaptive connectivity phenotypes [20,28]. In this context, mechanistic biomarkers may support mechanism-based patient stratification and facilitate the development of more targeted neuromodulation strategies.

Despite this conceptual complementarity, biomarker research in SCS remains highly fragmented. Most studies evaluate single modalities in isolation, without integrating electrophysiological, imaging, and molecular data. As a result, current approaches fail to capture the multidimensional nature of treatment response and limit translational applicability.

**Figure 2:**
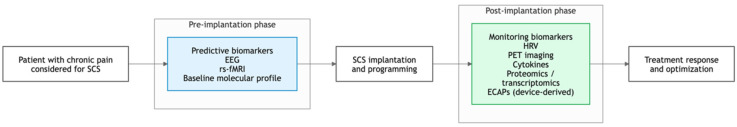
Proposed multimodal biomarker framework in spinal cord stimulation (SCS). A two-stage model integrating predictive and monitoring biomarkers. Baseline biomarkers obtained during the pre-implantation phase are used for patient stratification, while dynamic biomarkers assessed after implantation—including device-derived signals such as ECAPs—enable objective monitoring and optimization of treatment response.

To address this gap, a multimodal biomarker framework can be proposed, integrating predictive and monitoring domains within a unified clinical model (Figure 2). This approach involves a two-stage process: an initial pre-implantation phase, in which baseline biomarkers are used for patient stratification, followed by a post-implantation phase, where dynamic biomarkers are used to monitor and characterize treatment response over time. Such a model is consistent with established principles in biomarker development, combining baseline risk assessment with longitudinal evaluation of therapeutic effects [34,35], and supported by clinical perspectives in neuromodulation [36]. This framework should be interpreted as a conceptual model requiring prospective validation.

Beyond biomarker discovery, successful clinical translation will require demonstration of analytical validity, clinical validity, and clinical utility across independent cohorts. In addition to predictive performance, future biomarker strategies should address reproducibility, cost-effectiveness, regulatory considerations, and feasibility of integration into routine neuromodulation workflows. The absence of standardized validation pathways remains a major barrier to converting exploratory biomarkers into clinically actionable tools.

The implementation of multimodal strategies will require standardized acquisition protocols, larger multicentre datasets, and advanced analytical approaches, including machine learning and data integration techniques [9,37]. Ultimately, the integration of complementary biomarker domains may support a transition from empirical decision-making toward mechanism-based and personalized neuromodulation strategies.

## 6. Current Limitations

Several limitations should be considered when interpreting the current evidence on biomarkers in spinal cord stimulation (SCS).

The available literature is predominantly based on small, single-center cohorts, which limits statistical power and generalizability. Most studies include fewer than 30 patients, increasing the risk of overfitting. This limitation is particularly relevant in studies applying machine learning techniques to high-dimensional biomarker data, where model performance may not generalize across independent cohorts [11,21,23,24].

In addition, there is substantial heterogeneity across studies in terms of patient populations, pain aetiologies, stimulation paradigms, and outcome definitions. This variability complicates comparisons between studies and hinders the identification of standardized biomarker thresholds or clinically applicable cut-offs. An additional challenge is the potential paradigm-specific nature of biomarker responses across different SCS modalities, including tonic, burst, high-frequency, and closed-loop stimulation. These paradigms may engage distinct neural networks and physiological mechanisms, limiting the generalizability of biomarker findings across stimulation approaches and complicating the development of universal biomarker models.

Methodological variability further limits reproducibility. Differences in signal acquisition and processing for electrophysiological data, variability in imaging protocols, and lack of standardization in molecular analyses introduce inconsistencies that may affect external validity. For molecular and omics-based biomarkers, additional sources of variability include pre-analytical sample handling, assay reproducibility, inter-platform differences, normalization strategies, and bioinformatic processing pipelines, all of which may affect reproducibility across centers.

Another important limitation is the predominance of single-modality approaches. Most studies evaluate individual biomarker domains in isolation, without integrating electrophysiological, imaging, and molecular data. This reductionist perspective limits the ability to capture the multidimensional nature of SCS response and may underestimate the potential of multimodal strategies [13,21,22,24,31].

Furthermore, most studies focus on short-term outcomes, with limited longitudinal follow-up [10,11,12,13,15,21,22]. As a result, the temporal stability in controlled clinical settings of biomarker signals and their relationship with long-term clinical outcomes remain insufficiently characterized.

Finally, although associations between biomarkers and clinical outcomes have been reported, there is a lack of external validation and prospective biomarker-driven trials. To date, no study has demonstrated that biomarker-based stratification improves clinical decision-making or patient outcomes compared with standard care.

## 7. Future Directions

Future research in spinal cord stimulation (SCS) biomarkers should focus on overcoming current limitations through the development of integrated, multimodal, and clinically applicable approaches.

A primary priority is the design of multimodal biomarker models that combine electrophysiological, neuroimaging, and molecular data within unified analytical frameworks. The integration of heterogeneous data sources has demonstrated potential to improve predictive performance in other areas of biomedical research, suggesting a potential application in neuromodulation [38]. In the context of SCS, such approaches may enable more accurate patient stratification and a better characterization of response phenotypes.

As an example of a testable multimodal strategy, future studies could evaluate whether baseline rs-fMRI connectivity patterns combined with intraoperative EEG responses improve prediction of SCS outcomes beyond either modality alone. In this hypothetical model, baseline functional connectivity could capture pre-existing network susceptibility to neuromodulation, whereas intraoperative EEG changes could reflect early neurophysiological engagement during stimulation. A prospective study comparing single-modality and combined models using predefined responder criteria and external validation would help determine whether multimodal integration provides incremental predictive value.

The application of advanced analytical techniques, including machine learning and data fusion strategies, represents another key direction. While preliminary studies have demonstrated the feasibility of machine learning-based prediction using single-modality data [21], future efforts should aim to develop robust, externally validated models incorporating multiple biomarker domains. This will require larger datasets, standardized acquisition protocols, and multicenter collaboration.

Another promising avenue is the development of biomarker-driven clinical trials, in which patient selection, treatment allocation, or therapy optimization are guided by objective biological measures. Established frameworks for biomarker development emphasize the importance of progressing from exploratory associations toward prospective validation and clinical utility [39]. In SCS, such trials are currently lacking and represent a critical step toward translation.

In parallel, the emergence of closed-loop neuromodulation systems offers an opportunity to integrate biomarkers directly into therapeutic control. Systems based on physiological feedback signals, such as evoked compound action potentials (ECAPs), provide real-time information on dorsal column activation and enable adaptive modulation of stimulation parameters. Unlike traditional predictive biomarkers, ECAPs do not reflect baseline biological susceptibility but rather provide real-time physiological feedback on neural activation during stimulation. In this context, ECAPs may be conceptualized as dynamic functional biomarkers, enabling continuous monitoring of therapy delivery and adaptive optimization of stimulation parameters. Within the proposed framework, ECAPs should be considered a subtype of monitoring biomarkers rather than a distinct fourth category, because they provide real-time information on stimulation-induced neural activation and support ongoing therapy optimization rather than pre-treatment patient stratification. This distinguishes them from baseline predictive biomarkers while supporting their integration within longitudinal biomarker-guided neuromodulation frameworks. Clinical evidence from industry-sponsored studies suggests that ECAP-controlled stimulation may maintain more consistent neural activation and improve therapeutic stability compared with conventional open-loop approaches [30,40]. The incorporation of such device-derived biomarkers may represent an important step toward personalized and responsive neuromodulation strategies within precision medicine frameworks [41]. Further independent validation is required to establish their broader applicability as functional biomarkers.

Finally, future research should aim to establish standardized protocols and reporting guidelines for biomarker acquisition, analysis, and validation in SCS. Harmonization across studies will be essential to improve reproducibility, facilitate data sharing, and accelerate the translation of biomarker research into clinical practice.

## 8. Conclusions

Biomarker research in spinal cord stimulation (SCS) has expanded across electrophysiological, neuroimaging, and molecular domains, providing important insights into the mechanisms underlying neuromodulation. However, the clinical translation of these findings remains limited.

Among available approaches, electrophysiological and neuroimaging biomarkers appear to hold promise for patient stratification, whereas autonomic, metabolic, and immunological markers provide objective measures of treatment-induced changes. Nonetheless, most findings are derived from small, single-center studies and lack external validation.

A major limitation of the current field is the fragmentation of biomarker research, with most studies focusing on single modalities in isolation. The integration of predictive and monitoring biomarkers into multimodal frameworks represents a key step toward improving clinical applicability.

Future research should prioritize standardized methodologies, multicenter validation, and biomarker-driven clinical trials. The incorporation of adaptive neuromodulation systems and device-derived signals, such as ECAPs, may further facilitate the transition toward personalized and mechanism-based neuromodulation strategies.

## Data Availability

No new data were created or analyzed in this study.

## References

[1] Goldberg D.S., McGee S.J. (2011). Pain as a global public health priority. BMC Public Health.

[2] Treede R.D., Rief W., Barke A., Aziz Q., Bennett M.I., Benoliel R., Cohen M., Evers S., Finnerup N.B., First M.B. (2019). Chronic pain as a symptom or a disease: The IASP Classification of Chronic Pain for the International Classification of Diseases (ICD-11). Pain.

[3] Deer T.R., Mekhail N., Provenzano D., Pope J., Krames E., Leong M., Levy R.M., Abejon D., Buchser E., Burton A. (2014). The appropriate use of neurostimulation of the spinal cord and peripheral nervous system for the treatment of chronic pain and ischemic diseases: The Neuromodulation Appropriateness Consensus Committee. Neuromodulation.

[4] Kumar K., Taylor R.S., Jacques L., Eldabe S., Meglio M., Molet J., Thomson S., O’Callaghan J., Eisenberg E., Milbouw G. (2007). Spinal cord stimulation versus conventional medical management for neuropathic pain: A multicentre randomised controlled trial in patients with failed back surgery syndrome. Pain.

[5] Kapural L., Yu C., Doust M.W., Gliner B.E., Vallejo R., Sitzman B.T., Amirdelfan K., Morgan D.M., Brown L.L., Yearwood T.L. (2015). Novel 10-kHz High-frequency Therapy (HF10 Therapy) Is Superior to Traditional Low-frequency Spinal Cord Stimulation for the Treatment of Chronic Back and Leg Pain: The SENZA-RCT Randomized Controlled Trial. Anesthesiology.

[6] Deer T.R., Grider J.S., Lamer T.J., Pope J.E., Falowski S., Hunter C.W., Provenzano D.A., Slavin K.V., Russo M., Carayannopoulos A. (2020). A Systematic Literature Review of Spine Neurostimulation Therapies for the Treatment of Pain. Pain Med..

[7] Shanthanna H., Eldabe S., Provenzano D.A., Bouche B., Buchser E., Chadwick R., Doshi T.L., Duarte R., Hunt C., Huygen F. (2023). Evidence-based consensus guidelines on patient selection and trial stimulation for spinal cord stimulation therapy for chronic non-cancer pain. Reg. Anesth. Pain Med..

[8] Eldabe S., Duarte R.V., Gulve A., Thomson S., Baranidharan G., Houten R., Jowett S., Sandhu H., Chadwick R., Brookes M. (2020). Does a screening trial for spinal cord stimulation in patients with chronic pain of neuropathic origin have clinical utility and cost-effectiveness (TRIAL-STIM)? A randomised controlled trial. Pain.

[9] Mackey S., Aghaeepour N., Gaudilliere B., Kao M.C., Kaptan M., Lannon E., Pfyffer D., Weber K. (2025). Innovations in acute and chronic pain biomarkers: Enhancing diagnosis and personalized therapy. Reg. Anesth. Pain. Med..

[10] Poply K., Haroon A., Ganeshan B., Nikolic S., Sharma S., Ahmad A., Ellamushi H., Parsai A., Mehta V. (2023). Dynamic Brain Imaging Response to Spinal Cord Stimulation Differential Frequencies DiFY SCS-PET Clinical Trial. Neuromodulation.

[11] Ueno K., Oshiro Y., Kan S., Nomura Y., Satou H., Obata N., Mizobuchi S. (2025). Resting-state brain functional connectivity in patients with chronic intractable pain who respond to spinal cord stimulation therapy. Br. J. Anaesth..

[12] Goudman L., Brouns R., Linderoth B., Moens M. (2021). Effects of Spinal Cord Stimulation on Heart Rate Variability in Patients with Failed Back Surgery Syndrome: Comparison Between a 2-lead ECG and a Wearable Device. Neuromodulation.

[13] Kogias S.S., O’Brien J.A., Robertson R.V., Peng A., Tinoco-Mendoza F.A., Ramachandran A., Henderson L.A., Austin P.J. (2025). 10-kHz High-Frequency Spinal Cord Stimulation Significantly Reduces Proinflammatory Cytokines and Distinct Populations of T Lymphocytes in Patients with Persistent Spinal Pain Syndrome Type 2. Neuromodulation.

[14] Fabregat-Cid G., Cedeño D.L., Harutyunyan A., Rodríguez-López R., Monsalve-Dolz V., Mínguez-Martí A., Hernández-Cádiz M.J., Escrivá-Matoses N., Villanueva-Pérez V., Asensio Samper J.M. (2023). Effect of Conventional Spinal Cord Stimulation on Serum Protein Profile in Patients with Persistent Spinal Pain Syndrome: A Case-Control Study. Neuromodulation.

[15] Fabregat-Cid G., Cedeno D.L., De Andrés J., Harutyunyan A., Monsalve-Dolz V., Mínguez-Martí A., Escrivá-Matoses N., Asensio-Samper J.M., Carnaval T., Villoria J. (2025). Insights into the pathophysiology and response of persistent spinal pain syndrome type 2 to spinal cord stimulation: A human genome-wide association study. Reg. Anesth. Pain. Med..

[16] De Andrés J., Navarrete-Rueda F., Fabregat G., García-Gutiérrez M.S., Monsalve-Dolz V., Harutyunyan A., Mínguez-Martí A., Rodriguez-Lopez R., Manzanares J. (2021). Differences in Gene Expression of Endogenous Opioid Peptide Precursor, Cannabinoid 1 and 2 Receptors and Interleukin Beta in Peripheral Blood Mononuclear Cells of Patients with Refractory Failed Back Surgery Syndrome Treated with Spinal Cord Stimulation: Markers of Therapeutic Outcomes?. Neuromodulation.

[17] Zebhauser P.T., Hohn V.D., Ploner M. (2023). Resting-state electroencephalography and magnetoencephalography as biomarkers of chronic pain: A systematic review. Pain.

[18] Kupers R., Kehlet H. (2006). Brain imaging of clinical pain states: A critical review and strategies for future studies. Lancet Neurol..

[19] Puk O., Jabłońska M., Sokal P. (2023). Immunomodulatory and endocrine effects of deep brain stimulation and spinal cord stimulation—A systematic review. Biomed. Pharmacother..

[20] Chakravarthy K.V., Xing F., Bruno K., Kent A.R., Raza A., Hurlemann R., Kinfe T.M. (2019). A Review of Spinal and Peripheral Neuromodulation and Neuroinflammation: Lessons Learned Thus Far and Future Prospects of Biotype Development. Neuromodulation.

[21] Gopal J., Bao J., Harland T., Pilitsis J.G., Paniccioli S., Grey R., Briotte M., McCarthy K., Telkes I. (2025). Machine learning predicts spinal cord stimulation surgery outcomes and reveals novel neural markers for chronic pain. Sci. Rep..

[22] Kinfe T.M., Muhammad S., Link C., Roeske S., Chaudhry S.R., Yearwood T.L. (2017). Burst Spinal Cord Stimulation Increases Peripheral Antineuroinflammatory Interleukin 10 Levels in Failed Back Surgery Syndrome Patients with Predominant Back Pain. Neuromodulation.

[23] Telkes L., Hancu M., Paniccioli S., Grey R., Briotte M., McCarthy K., Raviv N., Pilitsis J.G. (2020). Differences in EEG patterns between tonic and high frequency spinal cord stimulation in chronic pain patients. Clin. Neurophysiol..

[24] Witjes B., Starmans M.P.A., Huygen F., de Vos C.C. (2025). Classification of chronic pain and spinal cord stimulation response using machine learning in magnetoencephalography data. PLoS ONE.

[25] FDA-NIH Biomarker Working Group (2016). BEST (Biomarkers, EndpointS, and Other Tools) Resource.

[26] Strimbu K., Tavel J.A. (2010). What are biomarkers?. Curr. Opin. HIV AIDS.

[27] Fiúza-Fernandes J., Pereira-Mendes J., Esteves M., Radua J., Picó-Pérez M., Leite-Almeida H. (2025). Common neural correlates of chronic pain—A systematic review and meta-analysis of resting-state fMRI studies. Prog. Neuropsychopharmacol. Biol. Psychiatry.

[28] De Ridder D., Vanneste S., Smith M., Adhia D. (2022). Pain and the Triple Network Model. Front. Neurol..

[29] Ferraro S., Klugah-Brown B., Tench C.R., Yao S., Nigri A., Demichelis G., Pinardi C., Bruzzone M.G., Becker B. (2022). Dysregulated anterior insula reactivity as robust functional biomarker for chronic pain-Meta-analytic evidence from neuroimaging studies. Hum. Brain Mapp..

[30] Mekhail N., Levy R.M., Deer T.R., Kapural L., Li S., Amirdelfan K., Hunter C.W., Rosen S.M., Costandi S.J., Falowski S.M. (2020). Long-term safety and efficacy of closed-loop spinal cord stimulation to treat chronic back and leg pain (Evoke): A double-blind, randomised, controlled trial. Lancet Neurol..

[31] De Andres J., Ten-Esteve A., Harutyunyan A., Romero-Garcia C.S., Fabregat-Cid G., Asensio-Samper J.M., Alberich-Bayarri A., Marti-Bonmati L. (2021). Predictive Clinical Decision Support System Using Machine Learning and Imaging Biomarkers in Patients with Neurostimulation Therapy: A Pilot Study. Pain Physician.

[32] Tracey I., Bushnell M.C. (2009). How neuroimaging studies have challenged us to rethink: Is chronic pain a disease?. J. Pain.

[33] Kucyi A., Davis K.D. (2015). The dynamic pain connectome. Trends Neurosci..

[34] Hoang K.B., Turner D.A. (2019). The Emerging Role of Biomarkers in Adaptive Modulation of Clinical Brain Stimulation. Neurosurgery.

[35] Dunn G., Emsley R., Liu H., Landau S. (2013). Integrating biomarker information within trials to evaluate treatment mechanisms and efficacy for personalised medicine. Clin. Trials.

[36] Knotkova H., Hamani C., Sivanesan E., Le Beuffe M.F.E., Moon J.Y., Cohen S.P., Huntoon M.A. (2021). Neuromodulation for chronic pain. Lancet.

[37] Ounajim A., Billot M., Goudman L., Louis P.Y., Slaoui Y., Roulaud M., Bouche B., Page P., Lorgeoux B., Baron S. (2021). Machine Learning Algorithms Provide Greater Prediction of Response to SCS Than Lead Screening Trial: A Predictive AI-Based Multicenter Study. J. Clin. Med..

[38] Karczewski K.J., Snyder M.P. (2018). Integrative omics for health and disease. Nat. Rev. Genet..

[39] Califf R.M. (2018). Biomarker definitions and their applications. Exp. Biol. Med..

[40] Russo M., Brooker C., Cousins M.J., Taylor N., Boesel T., Sullivan R., Holford L., Hanson E., Gmel G.E., Shariati N.H. (2020). Sustained Long-Term Outcomes with Closed-Loop Spinal Cord Stimulation: 12-Month Results of the Prospective, Multicenter, Open-Label Avalon Study. Neurosurgery.

[41] Ashley E.A. (2015). The precision medicine initiative: A new national effort. JAMA.

